# Changes in Cardiorespiratory Fitness and Probability of Developing Abdominal Obesity at One and Two Years

**DOI:** 10.3390/ijerph20064754

**Published:** 2023-03-08

**Authors:** Ricardo Ortega, Gonzalo Grandes, María Teresa Agulló-Ortuño, Sagrario Gómez-Cantarino

**Affiliations:** 1 Santa Barbara Primary, Care Centre, Servicio de Salud de Castilla-La Mancha, Av. de Santa Bárbara, 1, 45006 Toledo, Spain; ricardo.ortega3@alu.uclm.es; 2 Primary Care Research Unit of Bizkaia, Osakidetza Servicio Vasco de Salud, María Díaz de Haro, 58, 48010 Bilbao, Spain; 3 Department of Nursing, Physiotherapy and Occupational Therapy, Faculty of Physiotherapy and Nursing, University of Castilla-La Mancha, (UCLM), Avda. Carlos III, s/n, 45071 Toledo, Spain; 4 Health Sciences Research Unit: Nursing (UICISA: E), Coimbra School of Nursing (ESEnfC), 3004-011 Coimbra, Portugal

**Keywords:** cardiorespiratory fitness, cumulative incidence, health correlates, obesity, primary health care, waist circumference

## Abstract

Low cardiorespiratory fitness (CRF) is associated with an increased risk of developing abdominal obesity (AO), but it is not known if and/or how changes in CRF affect AO. We examined the relationship between changes in CRF and the risk of developing AO. This is a retrospective observational study of a cohort of 1883 sedentary patients, who had participated in a clinical trial of physical activity promotion carried out in Spain (2003–2007). These data were not used in the clinical trial. At baseline, they were free of cardiovascular disease, hypertension, diabetes, dyslipidemia, and/or AO; with an indirect VO_2_max measurement; 19–80 years old; and 62% were women. All the measures were repeated at 6, 12, and 24 months. The exposure factor was the change in CRF at 6 or 12 months, categorized in these groups: unfit-unfit, unfit-fit, fit-unfit, and fit-fit. We considered fit and unfit participants as those with VO_2_max values in the high tertile, and in the moderate or low tertiles, respectively. The main outcome measure was the risk of developing AO at one and two years, as defined by waist circumference >102 (men) and >88 (women) cm. At two years, 10.5% of the participants had developed AO: 13.5% in the unfit-unfit group of change at 6 months; 10.3% in the unfit-fit group (adjusted odds ratio (AOR) 0.86; 95% confidence interval (CI) 0.49–1.52); 2.6% in the fit-unfit group (AOR 0.13; 95%CI 0.03–0.61); and 6.0% in the fit-fit group (AOR 0.47; 95%CI 0.26–0.84). Those who stayed fit at 6 months decreased the probability of developing abdominal obesity at two years.

## 1. Introduction

Abdominal obesity (AO) is a greater health problem than general obesity (Body Mass Index ≥ 30 kg/m^2^) because it has a higher prevalence, a higher annual increase [1,2], and is a better predictor of obesity-related metabolic disorders [3] and the risk of cardiovascular disease (CVD) mortality [4].

Cardiorespiratory fitness (CRF) reflects the ability of the oxygen transport system to deliver oxygen to the muscles when they are performing physical work. The more physical work that is completed, the more oxygen is needed and the more the oxygen transport system has to work to meet those needs. This makes the organs involved in this transport improve their performance, and therefore translates into a higher CRF [5].

This higher CRF is also influenced by genetics in the early stages of life. After these stages, if physical work is not performed, it is not possible to maintain or increase that higher CRF [6]. Age, sex, and the presence of diseases or medication that influence the oxygen transport system also influence the CRF [5].

Only three studies about the relationship between CRF and abdominal obesity in adults have been detected in the scientific literature [7,8,9]. Two of them are cross-sectional observational studies that looked at the association between CRF and abdominal obesity [7,8]. The third is a longitudinal observational study that analyzed the relationship between CRF at a given time and the probability of developing abdominal obesity at two years [9]. This study about the relationship between CRF and obesity measured by different methods showed a 129% higher probability of developing AO in subjects with low CRF in comparison with those with high CRF. Furthermore, it relied on a single baseline assessment of CRF, with subsequent follow-up for AO development, but there are people who experience changes in their lifestyle that lead them to increase their CRF. So, it assumed that subjects were going to remain in the same condition during the observation follow-up period. With this single exposure assessment, it is difficult to discount the hypothesis that genetic factors or other confounder variables are an important cause of the relationship between CRF and AO. Moreover, changes in the variable of interest during follow-up may affect the subsequent probability of developing AO.

One way to examine this issue more completely is to evaluate the effect of changes in CRF on the probability of developing AO. The follow-up in the original study, PEPAF [10], provides an opportunity to evaluate the relationship of changes in CRF with AO in a cohort of men and women with four clinical evaluations over a two-year period.

Therefore, the hypothesis that changes in CRF at six and twelve months produced changes in the probability of developing AO at one and two years was retrospectively examined.

## 2. Materials and Methods

### 2.1. Design and Data Collection

This retrospective longitudinal observational study used data from the cohort of 4927 sedentary men and women (they did not meet the recommended aerobic physical activity levels [11] gathered between 2003 and 2004 for the PEPAF study and belonging to 11 Spanish health centers. The participants were between 19 and 80 years old and had no known cardiovascular disease. This cohort underwent a baseline evaluation and was re-evaluated at 6, 12, and 24 months. More details have been published elsewhere [10,12,13].

The PEPAF study complied with the guidelines of the Declaration of Helsinki. Its protocol was approved by the Institutional Clinical Research Ethics Committees (CRECs) for all of the participating centers (ClinicalTrials.gov Identifier: NCT00131079). Written informed consent was obtained from all participants involved in the study and patients signed a consent form before the baseline measurement.

### 2.2. Population

For the present study, we included participants who did not have hypertension, diabetes, or dyslipidemia (n = 2974). We excluded those who had missing values for oxygen uptake measurement (VO_2_max) and waist circumference (WC) at baseline (n = 218), as well as those men and women (n = 873) who already had a baseline WC > 102 cm and >88 cm, respectively. Thus, the cohort of this study included 1883 participants.

### 2.3. Measurements

For the purposes of this study, we selected the following socio-demographic and behavioral variables and measurements from the original study corresponding to baseline and 6- and 12-month visits: WC, VO_2_max, gender, age, smoking and alcohol habits, and physical activity levels. We also selected the measurement of WC at 24 months.

With the participant lying on the examination table, WC was measured level with the umbilicus, using a laminated meter tape around the uncovered abdomen.

VO_2_max was indirectly estimated by using a sub-maximal cycle ergometer (VarioBike 500) exercise test, using the YMCA-ACSM protocol, and was standardized by age, sex, and resting heart rate [14]. To predict VO_2_max according to the YMCA protocol, the participant pedaled on a cycle ergometer at specified kgm/min work rates for two to four 3 min stages, until his steady state heart rate rose to between 110 and 150 beats/min for two consecutive stages. Heart rate was recorded during the final 15 to 30 s of the second and third minutes.

Gender, age, social class, educational levels, employment status, physical activity levels (minutes per week and METs × hours per week spent in moderate or vigorous leisure and occupational activity in the week previous to the interview), tobacco (current smoker and non-smoker), and alcohol habits (drinker at no risk and drinker at risk) were recorded by questionnaires as explained in detail in the PEPAF study publication [13].

Nurses were trained for the performance of all measurements and for the guarantee of data quality. For data quality, a pilot study was conducted followed by a three-day review training, and double data entry into a centralized Oracle™ database. Quality control consisted of daily online supervision of the study process and data, daily feedback to nurses, monthly progress reports, and regular meetings with the collaborating investigators and nurses every four months.

### 2.4. Exposure Variable

The 1883 subjects of this sample were categorized as low, moderate, and high CRF according to the tertiles of their estimated VO_2_max and gender at baseline. These CRF tertiles were automatically generated by the statistical package, corresponding to the following VO_2_max values: low < 28.88 (men) and <21.94 (women) mL/kg/min, moderate from 28.88 to 35.71 (men) and 21.94 to 26.25 (women) mL/kg/min, and high > 35.71 (men) and > 26.25 (women) mL/kg/min. We obtained the number of METs corresponding to the value of each tertile by dividing those values of VO_2_max by 3.5 mL/kg/min. They were “low” < 8.25 (men) and <6.268 (women) METs, “moderate” from 8.25 to 10.21 (men) and 6.268 to 7.5 (women) METs, and “high” > 10.21 (men) and >7.5 (women) METs. Because in the previous study [9], low and moderate tertiles had a higher probability of developing AO than the high tertile, for the present study we now considered as unfit those subjects in the low and moderate tertiles (VO_2_max ≤ 35.71 mL/kg/min or ≤10.21 METs in men and ≤26.25 mL/kg/min or ≤7.5 METs in women), and as fit those subjects in the high tertile (VO_2_max > 35.71 mL/kg/min or >10.21 METs in men and >26.25 mL/kg/min or >10.21 METs in women). Using those VO_2_max values, unfit and fit subjects were classified at 6 and 12 months, according to the VO_2_max values obtained at the two follow-up visits.

Then, 4 groups of CRF change at 6 and 12 months were established, and they constituted the exposition groups, as follows: the group of unfit subjects at baseline who remained unfit at 6 or 12 months (unfit-unfit group); group of unfit subjects at baseline who became fit at 6 or 12 months (unfit-fit group); group of fit subjects at baseline who became unfit at 6 or 12 months (fit-unfit group); and group of fit subjects at baseline who remained fit at 6 or 12 months (fit-fit group). Each of the 3 groups (unfit-fit, fit-unfit, and fit-fit groups) was compared to unfit-unfit subjects as the reference group. The four groups obtained at 6 and 12 months were examined in separate models.

### 2.5. Outcome Variables

The cumulative incidence of AO, defined as the transition from a WC of ≤102 cm in men or ≤88 cm in women at the study baseline to a WC of >102 or 88 cm in men and women [15,16,17], respectively, was observed one or two years later.

Given that gender, age, social class, educational levels, employment status, changes in smoking and alcohol habits, and changes in physical activity levels may influence CRF [5] and/or abdominal fat, they were considered as potential confounders.

### 2.6. Data Analysis

All analyses were conducted using STATA. Means (SDs) were calculated for age, difference in WC, and difference in physical activity levels, and the percentage of participants in each category was determined for gender, social class, educational levels, employment status, AO, CRF, and smoking and alcohol habits. Those values were distributed according to the four groups of change in CRF and were compared using a chi-square test for the proportions of categorical variables and analysis of variance for the means of continuous variables. The one- and two-year cumulative incidences of AO were calculated by dividing the number of new cases at those points in time by the number of exposed participants in each of the four groups of change in CRF at 6 or 12 months. The probability of developing AO was computed as the odd ratios (ORs) of the one- or two-year cumulative incidence in the three exposed groups, divided by the cumulative incidence in the non-exposed group, as a point of reference, and adjusted for potential confounding variables and change in WC at 6 or 12 months by using multivariate mixed logistic regression models.

## 3. Results

At 6 and 12 months, 406 and 557 participants, respectively, failed to attend the VO_2_max and/or WC measurement visit, with remaining available data for 1477 and 1326 participants, respectively. Their mean age was, respectively, 41.5 (SD, 13.1) and 42.5 (SD, 13.2) years, of which 61.8% and 61.3% were women. There were no significant differences between those with valid and missing values in gender at 6 months, as well as VO_2_max and distribution in tertiles at 6 and 12 months. There were significant differences in age (*p* < 0.001; those with missing values were 3.2 and 3.1 younger, respectively, at 6 and 12 months), gender (*p* < 0.05; more women (32.5%) than men (28%) at 12 months), and waist circumference (*p* < 0.001; those with missing values had 2 and 1.6 cm, respectively, less at 6 and 12 months). 

Participants’ characteristics across the four groups of change in CRF at 6 and 12 months are summarized in Table 1 and Table 2.

Table 1 shows that all tests for trends throughout the four groups of change in CRF at 6 months were significant (*p* < 0.05), except for gender, social class, and changes in physical activity levels at 6 months.

Table 2 shows that all tests for trends throughout the four groups of change in CRF at 12 months were significant (*p* < 0.05), except for gender, social class, and changes in WC, and in physical activity levels at 12 months.

The numbers of new cases of AO at one and two years were, respectively, 97 (cumulative incidence: 6.98; 95% confidence interval (95%CI): 5.70–8.45) and 135 (cumulative incidence: 10.5; 95%CI: 8.88–12.32).

Table 3 shows cumulative incidence rates at one and two years across unfit-unfit, unfit-fit, fit-unfit, and fit-fit groups of change at 6 and 12 months. Compared to those participants who remained unfit at 6 or 12 months, it shows that those who remained fit at 6 months had a lower incidence of AO at one and two years, and those participants who remained fit at 12 months had a lower incidence of AO at two years. Moreover, compared to those who remained unfit at 6 months, those participants who changed from fit at baseline to unfit at 6 months had a lower incidence of AO at two years.

Table 4 shows the results of the adjusted ORs of changes in CRF at 6 months and the development of AO in the next six and eighteen months, as well as changes in CRF at 12 months and the development of AO in the next twelve months. Compared to the unfit-unfit change group at 6 months, the fit-unfit and fit-fit change groups were associated with a lower probability (OR = 0.26; 95%CI, 0.07–0.99 and OR = 0.44; 95%CI, 0.22–0.84, respectively) of developing AO in the next six months (at one year). 

Compared to the unfit-unfit change group at 6 months, the fit-unfit and fit-fit change groups were associated with a lower probability (OR = 0.12; 95%CI, 0.02–0.58 and OR = 0.45; 95%CI, 0.25–0.83, respectively) of developing AO in the next eighteen months (at two years). 

Compared to the unfit-unfit change group at 12 months, the fit-unfit and fit-fit change groups were associated with a lower probability (OR = 0.33; 95%CI, 0.13–0.83, and OR = 0.37; 95%CI, 0.20–0.68, respectively) of developing AO in the next twelve months (at two years).

Moreover, the multivariate analysis yielded the following results. Women had a higher probability of developing AO at one (OR = 2.29; 95%CI, 1.31–4.00) and two years (OR = 1.90; 95%CI, 1.14–3.16) than men. A middle or high school education had a higher probability of developing AO at one year (OR = 2.25; 95%CI, 1.03–4.91) than less education. Retirement had a higher probability of developing AO at two years (OR = 2.56; 95%CI, 1.03–6.38) compared to working outside of the home. For every cm of increment in WC at 6 months there was a greater probability of developing AO at one year (OR = 1.26; 95%CI, 1.18–1.34) and two years (OR = 1.17; 95%CI, 1.11–1.24), and for the same change in WC at 12 months there was a greater probability of developing AO at two years (OR = 1.20; 95%CI, 1.15–1.16). For every minute per week increment of physical activity at 6 and 12 months, there was a lower probability of developing AO at two years (OR = 0.997; 95%CI, 0.995–0.999 and OR = 0.997; 95%CI, 0.995–0.998, respectively). 

Continuing to smoke at 6 or 12 months had a higher probability of developing AO at two years (OR = 1.71; 95%CI, 1.12–2.65 and OR = 1.79; 95%CI, 1.15–2.78, respectively) than ceasing to smoke, and beginning to smoke at 6 months had a higher probability of developing AO at two years (OR = 2.80; 95%CI, 1.26–6.22). Being a risky drinker at 6 months had a higher probability of developing AO at two years (OR = 4.13; 95%CI, 1.35–12.6) than remaining a non-risky drinker.

## 4. Discussion

Data of this cohort of patients free of CVD, hypertension, diabetes, and dyslipidemia who had consulted their primary care physicians in Spain indicate that those who stayed fit at 6 or 12 months had a lower probability of the subsequent development of AO at one and two years, when compared to those who remained unfit.

Particularly, compared with remaining unfit, staying fit at 6 months was associated with a 56% and 55% decrease, respectively, at one and two years, in the probability of developing AO; changing from fit to unfit at 6 months was associated with a 74% and 88% decrease, respectively, at one and two years.

On the other hand, compared with remaining unfit, staying fit at 12 months was associated with a 63% decrease in the probability of developing AO at two years; furthermore, changing from fit to unfit at 12 months was associated with a 67% decrease at two years.

The lower probability of developing AO in those who stayed fit is consistent with the recommendation made in a previous article [9] of increasing CRF to over 10.2 and 7.5 METs, respectively, in men and women. These CRF levels are fairly consistent with the levels of an existing meta-analysis [18] about CRF and all-cause mortality and cardiovascular events. This meta-analysis was conducted using thirty-three studies: twenty-three of which included only men, four of which included men and women, and six of which did not mention the gender of the participants. It considered low CRF those levels below 7.9 METs, intermediate CRF those levels between 7.9 and 10.8 METs, and high CRF those levels above 10.8 METs, but it did not distinguish between men and women.

Its most plausible explanation was that people who stay fit lead a lifestyle that involves physical efforts of sufficient intensity to maintain that fitness [5], and to maintain the physical efforts that require the utilization of energy from the body’s fats. The higher energy expenditure of people who stay fit may either generate a neutral (energy expenditure equals energy intake) or excess (energy expenditure exceeds energy intake) energy balance, thereby avoiding an excessive accumulation of fat in the abdomen.

The lower probability of developing AO in those who changed from fit to unfit was probably due to the short time elapsed since they changed from fit to unfit, which was not enough to increase the accumulation of fat in the abdomen until reaching AO. It also probably depends on how long they maintained a high level of CRF. A study about changes in CRF and mortality [19] found that those participants who changed from fit to unfit had a lower risk of mortality than those who stayed unfit.

The same can be said for those who changed from unfit to fit. The time elapsed since they changed from unfit to fit was probably insufficient to prevent them from continuing to accumulate fat in the abdomen.

Our search has not identified any study on the change in CRF and the development, not only of AO, but of any type of obesity. The existing studies on the change in CRF looked for its influence on general and cardiovascular mortality [19,20,21,22], cancer mortality [23], the risk of dementia incidence and mortality [24], the risk of stroke and death [25], the risk of atrial fibrillation and heart failure [26], or the risk of hypertension [27,28].

### 4.1. Limitations

The limitations of this study may include methods of measuring CRF and WC, the number of cases developed from AO, the short follow-up period, and a potential bias from loss to follow-up.

The exposure variable, CRF, was measured by an indirect method and a sub-maximal stress test, which are valid and feasible measuring ways for primary care [29], and were used in numerous studies, although the most accurate form of measurement is a direct method and a maximum exercise test [5].

The outcome variable, AO, was measured by waist circumference, instead of computerized tomography, because it is a valid and more feasible method of measurement for primary care and has been used in numerous studies [30].

Waist circumference was validated against other ways of measuring abdominal fat, such as computed tomography, and correlations of 0.84, 0.71, and 0.73 with total, subcutaneous, and visceral abdominal fat, respectively, have been found [31].

On the other hand, the number of cases of AO developed at one and two years according to the changes in CRF (unfit-unfit, unfit-fit, fit-unfit, and fit-fit) at 6 and 12 months were as follows: sixty-one, eleven, three, and fourteen new cases developed at one year for changes at 6 months; eighty-four, nineteen, two, and eighteen new cases developed at two years for changes at 6 months; and seventy-nine, seventeen, six, and sixteen new cases developed at two years for changes at 12 months. Note that the fit-unfit group of change in CRF at 6 months did not reach five cases at one year and two years. That situation, in addition to the fact that only 75 and 98 participants at 6 and 12 months, respectively, changed from fit to unfit, may have misrepresented the probability of developing AO in this group compared to that of the unfit-unfit. The incidences of the fit-unfit change group were the lowest of the three exposure groups.

Compared to what would be expected, the short follow-up period might have influenced the results of the unfit-fit and fit-unfit groups in the sense of having made possible other results with a longer follow-up period, such as a lesser incidence of AO in the unfit-fit group and a similar or higher incidence in the fit-unfit group compared with the unfit-unfit group.

Regarding losses, we think that biases were probably of little importance given that differences between those with valid and missing values were small.

### 4.2. Strengths

Possible strengths of the study are as follows: the repeated performed measures of exposure and the results over time in this study in which the results remained constant, although with different magnitudes at each moment of CRF changes and development of AO; the consideration of low and moderate CRF tertiles as unfit, which represent 66% of the cohort, and the high CRF tertile as fit, which represents only 33% of the cohort, which caused the fit-unfit group of CRF change to have the least number of participants of all the groups.

## 5. Conclusions

This study shows that staying fit over time protects against the development of AO. It also shows that staying fit and becoming unfit may still protect against the development of AO during a certain period of time, which will probably be in relation to the time that one remains fit. Having changed from unfit to fit might also protect against the development of AO but it surely needs a longer period of time. These last two conclusions require additional studies with longer time exposure to CRF changes. Furthermore, these results must be interpreted with caution.

Given that CRF is closely linked to the level of the person’s physical activity, the advice for patients would be to maintain the level of physical activity recommended by the international organizations for those patients who are fit, and to begin to meet those recommendations for those patients who are unfit, in order to avoid abdominal obesity.

## Figures and Tables

**Table 1:** Participants’ characteristics of sample individuals across the four groups of change in cardiorespiratory fitness at 6 months.

Variable	Unfit-Unfit n = 753	Unfit-Fit n = 243	Fit-Unfit n = 97	Fit-Fit n = 384	*p*
Gender					0.820
Female	458 (60.8%)	155 (63.8%)	62 (63.9%)	239 (62.2%)	
Male	295 (39.2%)	88 (36.2%)	35 (36.1%)	145 (37.8%)	
Age, years	46.9 (13.5) *	38.5 (9.6) *‡	38.9 (11.3) *↑	33.4 (9.0) *‡↑	<0.001
Social class					0.464
Manual worker	77 (10.2%)	28 (11.5%)	5 (5.1%)	40 (10.4%)	
Intermediate employee	94 (12.5%)	31 (12.8%)	19/19.6%)	47 (12.2%)	
Manager small enterprise	219 (29.1%)	78 (32.1%)	32 (33.0%)	120 (31.3%)	
Manager large enterprise	363 (48.2%)	106 (43.6%)	41 (42.3%)	177 (46.1%)	
Educational level					<0.001
None	177 (23.5%)	73 (30.0%)	33 (34.0%)	130 (33.9%)	
Elementary school	392 (52.1%)	149 (61.3%)	56 (57.7%)	232 (60.4%)	
Middle or high school	167 (22.2%)	19 (7.8%)	6 (6.2%)	22 (5.7%)	
University studies	17 (2.2%)	2 (0.8%)	2 (2.1%)	0	
Employment status					<0.001
Works outside of home	110 (14.6%)	7 (2.9%)	4 (4.1%)	3 (0.8%)	
Homemaker	441 58.6%)	179 (73.6%)	67 (69.1%)	275 (71.6%)	
Retired	126 (16.7%)	23 (9.5%)	9 (9.3%)	25 (6.5%)	
Student	40 (5.3%)	19 (7.8%)	10 (10.3%)	26 (6.8%)	
Unemployed	17 (2.3%)	9 (3.7%)	6 (6.2%)	43 (11.2%)	
Other	19 (2.5%)	6 (2.5%)	1 (1.0%)	12 (3.1%)	
dWC, cm	0.57 (3.3)	0.05 (3.2)	1.02 (3.9)	0.31 (2.8)	0.038
dVO_2_max, mL/kg/min	1.06 (3.3) *	7.06 (4.6) *‡	−7.15 (5.4) *‡↑	1.58 (6.2) ‡↑	<0.001
Physical activity levels					
dMETs-h/week	0.63 (2.4)	0.71 (2.0)	0.04 (3.3)	0.69 (3.3)	0.179
dMinutes/week	56.9 (241.1)	65.7 (221.5)	28.5 (380.8)	76.1 (302.0)	0.406
Changes in smoking status					<0.001
Continue without smoking	482 (64.0%)	140 (57.6%)	48 (49.5%)	191 (49.7%)	
Continue smoking	229 (30.4%)	87 (35.8%)	36 (37.1%)	169 (44.0%)	
Begin to smoke	29 (3.9%)	8 (3.3%)	11 (11.3%)	18 (4.7%)	
Stop smoking	13 (1.7%)	8 (3.3%)	2 (2.1%)	6 (1.6%)	
Changes in alcoholic status					0.012
Remain non-risky drinker	712 (94.6%)	222 (91.4%)	90 (92.8%)	336 (87.5%)	
Remain risky drinker	15 (2.0%)	9 (3.7%)	2 (2.1%)	25 (6.5%)	
Begin to be risky drinker	14 (1.9%)	7 (2.9%)	3 (3.1%)	12 (3.1%)	
Stop being risky drinker	12 (1.5%)	5 (2.0%)	2 (2.0%)	11 (2.9%)	

*, ‡, ↑ = significant differences between groups, *p* < 0.05. Data are presented as mean (SD) or n (%). Abbreviations: dWC, difference in waist circumference between baseline and 6-month follow-up visits; dVO_2_max, difference in maximal oxygen uptake between baseline and 6-month follow-up visits; dMETs-h/week, difference in METs-h/week between baseline and 6-month follow-up visits; dMinutes/week, difference in minutes/week between baseline and 6-month follow-up visits.

**Table 2:** Participants’ characteristics of sample individuals across the four groups of change in cardiorespiratory fitness at 12 months.

Variable	Unfit-Unfit n = 682	Unfit-Fit n = 216	Fit-Unfit n = 116	Fit-Fit n = 312	*p*
Gender					0.473
Female	414 (60.7%)	132 (61.1%)	79 (68.1%)	188 (60.3%)	
Male	268 (39.3%)	84 (38.9%)	37 (31.9%)	124 (39.7%)	
Age, years	48.1 (13.5) *	38.6 (9.3) *‡	38.6 (11.1) *↑	34.3 (9.0) *‡↑	<0.001
Social class					0.441
Manual worker	70 (10.3%)	26 (12.0%)	10 (8.6%)	27 (8.7%)	
Intermediate employee	77 (11.3%)	30 (13.9%)	13 (11.2%)	41 (13.1%)	
Manager small enterprise	193 (28.3%)	74 (34.3%)	36 (31.0%)	98 (31.4%)	
Manager large enterprise	342 (50.1%)	86 (39.8%)	57 (49,2%)	146 (46.8%)	
Educational level					<0.001
None	143 (21.0%)	71 (32.9%)	41 (35.4%)	99 (31.7%)	
Elementary school	366 (53.7%)	127 (58.8%)	66 (56.9%)	197 (63.2%)	
Middle or high school	155 (22.7%)	18 (8.3%)	7 (6.0%)	16 (5.1%)	
University studies	18 (2.6%)	0	2 (1.7%)	0	
Employment status					<0.001
Works outside of home	105 (15.4%)	5 (2.3%)	2 (1.7%)	3 (1.0%)	
Homemaker	390 (57.2%)	163 (75.5%)	81 (69.8%)	223 (71.5%)	
Retired	108 (15.8%)	27 (12.5%)	9 (7.8%)	25 (8.0%)	
Student	48 (7.0%)	10 (4.6%)	11 (9.5%)	24 (7.7%)	
Unemployed	15 (2.2%)	7 (3.2%)	9 (7.8%)	29 (9.3%)	
Other	16 (2.4%)	4 (1.9%)	4 (3.4%)	8 (2.5%)	
dWC, cm	0.60 (4.1)	0.46 (4.6)	1.07 (3.9)	0.60 (3.5)	0.626
dVO_2_max, mL/kg/min	0.60 (3.5) *	7.76 (5.4) *‡	−6.38 (4.3) *‡↑	1.30 (7.1) ‡↑	<0.001
Physical activity levels					
dMETs-h/week	0.95 (2.7)	1.01 (2.8)	1.00 (3.2)	0.63 (3.5)	0.368
dMinutes/week	81.8 (269.9)	83.9 (249.5)	88.5 (273.7)	46.1 (283.1)	0.214
Changes in smoking status					<0.001
Continue without smoking	448 (65.7%)	126 (58.4%)	60 (51.7%)	155 (49.7%)	
Continue smoking	183 (26.8%)	76 (35.2%)	38 (32.8%)	132 (42.3%)	
Begin to smoke	37 (5.4%)	7 (3.2%)	13 (11.2%)	22 (7.0%)	
Stop smoking	14 (2.1%)	7 (3.2%)	5 (4.3%)	3 (1.0%)	
Changes in alcoholic status					<0.001
Remain non-risky drinker	650 (95.3%)	194 (89.8%)	107 (92.2%)	279 (89.4%)	
Remain risky drinker	6 (0.9%)	6 (2.8%)	1 (0.9%)	17 (5.5%)	
Begin to be risky drinker	15 (2.2%)	11 (5.1%)	7 (6.0%)	10 (3.2%)	
Stop being risky drinker	11 (1.6%)	5 (2.3%)	1 (0.9%)	6 (1.9%)	

*, ‡, ↑ = significant differences between groups, *p* < 0.05. Data are presented as mean (SD) or n (%). Abbreviations: dWC, difference in waist circumference between baseline and 12-month follow-up visits; dVO_2_max, difference in maximal oxygen uptake between baseline and 12-month follow-up visits; dMETs-h/week, difference in METs-h/week between baseline and 12-month follow-up visits; dMinutes/week, difference in minutes/week between baseline and 12-month follow-up visits.

**Table 3:** Cumulative incidence rates of abdominal obesity across unfit-unfit, unfit-fit, fit-unfit, and fit-fit groups of change at 6 months and 12 months.

	**Changes in CRF at 6 Months**				
	**Unfit-Unfit**	**Unfit-Fit**	**Fit-Unfit**	**Fit-Fit**	***p***
Incidence at one year	9.20 (7.11–11.66)	5.12 (2.58–8.96)	3.41 (0.70–9.64)	4.24 (2.33–7.01)	0.008
Incidence at two years	13.50 (10.91–16.44)	10.33 (6.33–15.65)	2.67 (0.32–9.30)	6.06 (3.63–9.40)	0.001
	**Changes in CRF at 12 months**				
	**Unfit-Unfit**	**Unfit-Fit**	**Fit-Unfit**	**Fit-Fit**	***p***
Incidence at two years	13.01 (10.44–15.95)	9.60 (5.69–14.93)	6.12 (2.27–12.85)	5.90 (3.41–9.41)	0.006

Units are presented in percentage (95% confidence interval).

**Table 4:** Association of changes in cardiorespiratory fitness at six or twelve months with development of abdominal obesity at one or two years: multivariate adjusted odds ratios.

	AOR (95%CI)	*p*
One year		
Changes at 6 months ^a^		0.008
Unfit-Fit vs. Unfit-Unfit	0.55 (0.27–1.13)	
Fit-Unfit vs. Unfit-Unfit	0.26 (0.07–0.99)	
Fit-Fit vs. Unfit-Unfit	0.44 (0.22–0.84)	
Two years		
Changes at 6 months ^a^		<0.001
Unfit-Fit vs. Unfit-Unfit	0.87 (0.49–1.54)	
Fit-Unfit vs. Unfit-Unfit	0.12 (0.02–0.58)	
Fit-Fit vs. Unfit-Unfit	0.45 (0.25–0.83)	
Changes at 12 months ^b^		0.006
Unfit-Fit vs. Unfit-Unfit	0.67 (0.37–1.22)	
Fit-Unfit vs. Unfit-Unfit	0.33 (0.13–0.83)	
Fit-Fit vs. Unfit-Unfit	0.37 (0.20–0.68)	

Abbreviations: AOR, adjusted odds ratio; CI, confidence interval; *p*, probability. ^a^ Adjusted for gender, age, difference at 6 months in WC values and physical activity levels, as well as changes in smoking and alcohol status at 6 months. ^b^ Adjusted for gender, age, difference at 12 months in WC values and physical activity levels, as well as changes in smoking and alcohol status at 12 months.

## Data Availability

Data and code are available upon reasonable request. Data are only those corresponding to this article and come from the PEPAF study. Requests should be directed towards the corresponding author.
